# Complete Genome Sequences of Two Bacillus pumilus Strains from Cuatrociénegas, Coahuila, Mexico

**DOI:** 10.1128/genomeA.00364-18

**Published:** 2018-04-26

**Authors:** Eugenia Zarza, Luis D. Alcaraz, Bernardo Aguilar-Salinas, Africa Islas, Gabriela Olmedo-Álvarez

**Affiliations:** a Laboratorio de Biología Molecular y Ecología Microbiana, Departamento de Ingeniería Genética, Centro de Investigación y de Estudios Avanzados del Instituto Politécnico Nacional, Unidad Irapuato, Irapuato, Mexico; b Departamento de Biología Celular, Facultad de Ciencias, Universidad Nacional Autónoma de México, Mexico City, Mexico

## Abstract

We assembled the complete genome sequences of Bacillus pumilus strains 145 and 150a from Cuatrociénegas, Mexico. We detected genes codifying for proteins potentially involved in antagonism (bacteriocins) and defense mechanisms (abortive infection bacteriophage proteins and 4-azaleucine resistance). Both strains harbored prophage sequences. Our results provide insights into understanding the establishment of microbial interactions.

## GENOME ANNOUNCEMENT

Bacillus pumilus is a Gram-positive, spore-forming bacterium residing in soil and water and in some extreme environments. Some strains are resistant to high levels of radiation and oxidizing agents (1), and some antagonize other bacteria and disturb the structure and architecture of bacterial biofilms (e.g., Vibrio, Bacillus, and Pseudomonas [2–4]). Here, we report the complete genome sequence of B. pumilus 145 and B. pumilus 150a from the shallow water system of Churince in Cuatrociénegas, Coahuila, the detection of genes potentially involved in antagonism of other coinhabiting species, and the complete genome sequence of the Bacillus phage EZ-2018a. These findings will give insights into understanding the establishment of biological interactions. Total genomic DNA was sequenced with the PacBio RS II system, assembled with Canu v.1.3 (5), and circularized with Circlator v.1.3 (6). The B. pumilus 145 assembly resulted in two contigs representing the chromosome (3,937,399 bp, 265.81× mean coverage) and the Bacillus phage EZ-2018a (118,485 bp, 486.825× mean coverage), whereas the B. pumilus 150a assembly resulted in one contig (3,747,740 bp, 269.37× mean coverage). In the B. pumilus clade, the gene *gyrB* is the most informative to distinguish between closely related species (7). NCBI BLAST alignments (8) of *gyrB* showed 98% similarity between *B. pumilus* BAT47140 and B. pumilus 145 and 100% identity between B. pumilus WP_041816108 and B. pumilus 150a. We used Rapid Annotations using Subsystems Technology (RAST) (5) for genome annotation. Eight rRNA copies, 81 tRNAs, and 4,211 protein-coding sequences in 459 functional subsystems were identified in the B. pumilus 145 genome, and eight rRNA copies, 82 tRNAs, and 3,827 protein coding sequences in 466 subsystems in the B. pumilus 150a genome. There are 59 genes involved in virulence, disease, and defense in B. pumilus 145, and 61 in B. pumilus 150a. Twelve genes related to the bacteriocin stress response and one conferring tolerance to colicin E2 were annotated in both strains. Both strains harbored prophage sequences, as indicated by PHAST (9). B. pumilus 145 contained four intact prophage regions in the chromosome and the Bacillus phage EZ-2018a, whereas B. pumilus 150a harbored two intact prophage regions in its chromosome. A gene coding for an abortive infection bacteriophage resistance protein (*Abi*) belonging to the Abi_2 family was detected in one of the prophage regions of B. pumilus 145. This defense mechanism is a cell death system activated by phage infection and provides protection to the bacterial population by limiting viral replication. Top hits in a PSI-BLAST search (10) indicated that a similar gene (91% amino acid identity) was found in Bacillus safensis and in Bacillus subtilis (58% amino acid identity), but it was not present in any other B. pumilus sequenced genome, suggesting that B. pumilus 145 acquired *Abi* through a mobile genetic element. On the other hand, B. pumilus 150a contained *AzlC* and *AzlD* genes that codify for branched-chain amino acid transport proteins possibly involved in conferring resistance to 4-azaleucine—previously detected in other Bacillus genomes, but not in any other B. pumilus (11, 12). This leucine analogue is a potent growth inhibitor in several bacteria due to its incorporation into proteins in place of leucine (13, 14). 4-Azaleucine is produced by Streptomyces (15), one of the most abundant *Actinobacteria* in Cuatrociénegas (16).

### Accession number(s).

These genomes have been deposited in GenBank under the accession numbers CP027034, CP027116, and CP027117. The versions described in this paper are the first versions.
