# Correction for Zarza et al., “Complete Genome Sequences of Two Bacillus pumilus Strains from Cuatrociénegas, Coahuila, Mexico”

**DOI:** 10.1128/MRA.01253-18

**Published:** 2018-10-25

**Authors:** Eugenia Zarza, Luis D. Alcaraz, Bernardo Aguilar-Salinas, Africa Islas, Gabriela Olmedo-Álvarez

**Affiliations:** a Laboratorio de Biología Molecular y Ecología Microbiana, Departamento de Ingeniería Genética, Centro de Investigación y de Estudios Avanzados del Instituto Politécnico Nacional, Unidad Irapuato, Irapuato, Mexico; b Departamento de Biología Celular, Facultad de Ciencias, and Laboratorio Nacional de Ciencias de la Sostenibilidad (LANCIS), Instituto de Ecología, Universidad Nacional Autónoma de México, Mexico City, Mexico

## AUTHOR CORRECTION

Volume 6, no. 17, e00364-18, 2018, https://doi.org/10.1128/genomeA.00364-18. Page 1: The author affiliations should appear as shown in this correction.

